# Pigmented Spitz nevus of the lip

**DOI:** 10.1016/j.jdcr.2025.12.035

**Published:** 2025-12-29

**Authors:** João Teixeira, Mariana Pedroso, José Carlos Cardoso, Inês Coutinho, Hugo Schönenberger de Oliveira

**Affiliations:** Department of Dermatology and Venereology, Local Health Unit of Coimbra, Coimbra, Portugal

**Keywords:** dermoscopy, lip neoplasms, mucous membrane, pediatric, Spitz nevus

## Clinical presentation

**Fig 1:**
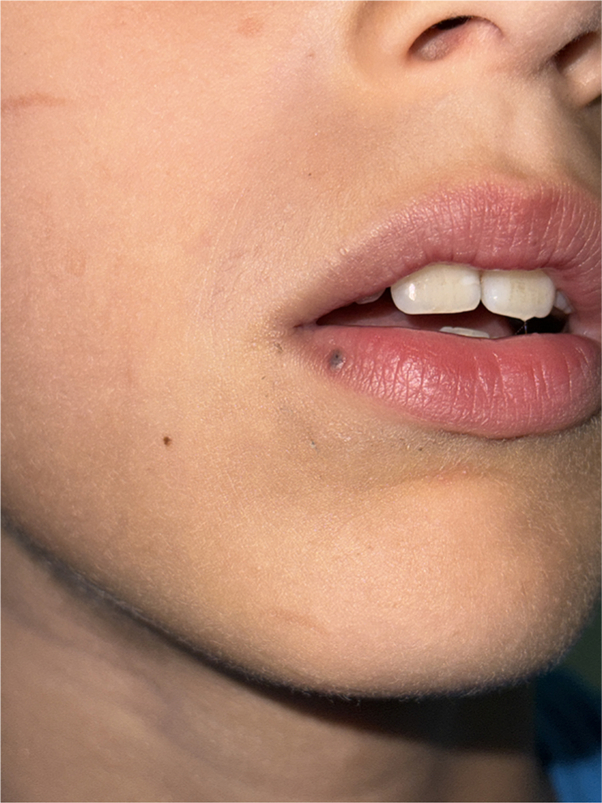
Clinical picture showing a 3-mm, well-circumscribed bluish papule with a smooth surface on the lower vermilion lip of a 7-year-old boy.

A 7-year-old boy presented with a solitary, asymptomatic pigmented papule on the lower vermilion lip that had been present for several months. Clinical examination revealed a 3-mm, well-circumscribed blue-gray papule with a smooth surface (Fig 1).

## Dermoscopic appearance

**Fig 2:**
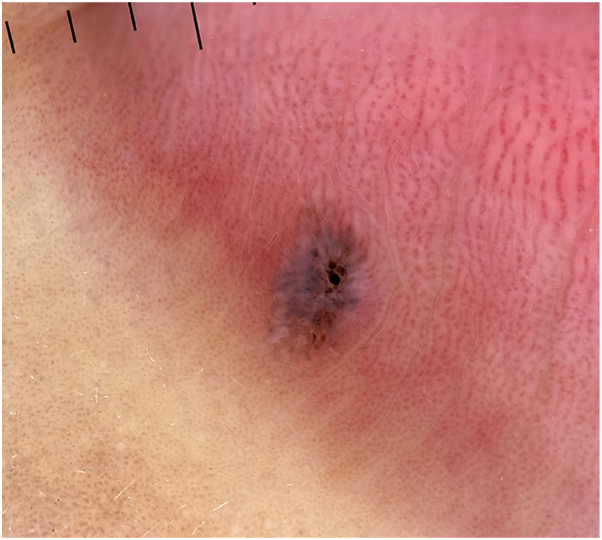
Polarized contact dermoscopy (Heine Delta One, 10×) demonstrating a structureless blue-gray background with central brown-black and blue globules radiating peripherally. The overall architecture is symmetrical and globular, producing a starburst-like appearance.

Dermoscopy showed a structureless blue-gray background with central brown-black dots and globules, blue globules radiating peripherally, and peripheral shiny white structures (Fig 2).

## Histologic diagnosis

**Fig 3:**
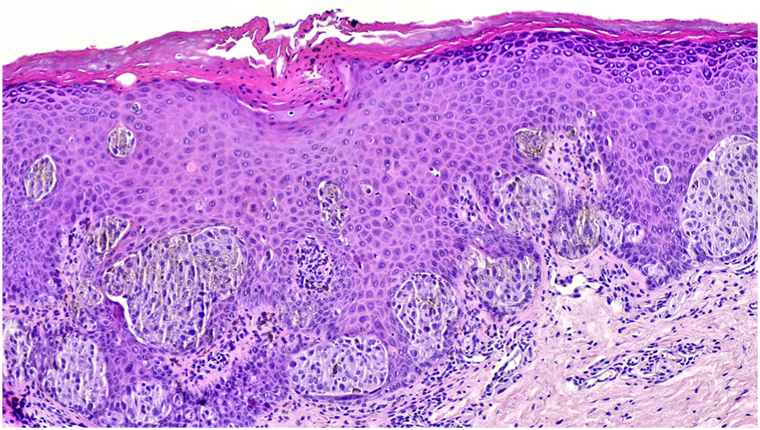
Histopathology (hematoxylin-eosin stain, 200×) showing epithelioid and spindle melanocytes at the dermoepidermal junction with no significant cytologic atypia, with clefts surrounding the nests, consistent with a junctional pigmented Spitz nevus. Focal pigmented parakeratosis was also noted.

Excisional biopsy revealed a symmetrical, well-circumscribed junctional melanocytic proliferation composed of epithelioid and spindle melanocytes arranged in nests at the dermoepidermal junction without significant cytologic atypia (Fig 3). These findings confirmed a junctional pigmented Spitz nevus.

Key message

Spitz nevi are uncommon melanocytic proliferations, representing about 1% of melanocytic nevi, typically arising in children and adolescents.^1^ They are classified as nonpigmented, appearing as pink to reddish papules, and pigmented Spitz nevi, presenting as brown to black macules or papules. Common sites include the extremities, face, and trunk.

Dermoscopically, pigmented Spitz nevi often display the starburst pattern, with radial streaks or pseudopods at the periphery and homogeneous central pigmentation.^1^ Other features include symmetrical streaks, regularly distributed dotted vessels, and homogeneous pigmentation. In this case, the globular pattern corresponded to the large junctional nests, and the grayish color was likely attributable to the overlying acanthotic epithelium. These features are consistent with those described in pigmented Spitz nevi at other cutaneous sites, although the lack of follicular structures and the prominent vascularity of the lip semimucosa may attenuate the globular pattern. Of note, the dermoscopic pattern in this case, although previously described in cutaneous pigmented Spitz nevi, has not, to our knowledge, been documented on the vermilion.

In fact, in mucosal locations such as the lip, Spitz nevi are exceedingly rare.^2^ In this context, traumatized angioma, venous lake, and melanotic labial macule should be considered in the differential diagnosis, particularly when typical dermoscopic features are absent, making histopathologic confirmation essential for accurate diagnosis.

## Conflicts of interest

None disclosed.
